# Salivary Metabolite Variation After High-Intensity Rowing Training and Potential Biomarker Screening for Exercise-Induced Muscle Damage

**DOI:** 10.3390/metabo15060405

**Published:** 2025-06-16

**Authors:** Yue Yi, Junjie Ding, Baoguo Wang, Yuxian Li, Liming Wang, Shumin Bo, Qiongqiong Ren, Aiqin Luo

**Affiliations:** 1 School of Life Science, Beijing Institute of Technology, Beijing 100081, China; yueyi@bit.edu.cn (Y.Y.); 3220242251@bit.edu.cn (J.D.); 3120221400@bit.edu.cn (B.W.); 2 Key Laboratory of Molecular Medicine and Biotherapy, Ministry of Industry and Information Technology, Beijing Institute of Technology, Beijing 100081, China; 3 School of Medical Technology, Beijing Institute of Technology, Beijing 100081, China; 6120180052@bit.edu.cn (Y.L.); limingw2008@163.com (L.W.); 4 College of Kinesiology and Health, Capital University of Physical Education and Sports, Beijing 100191, China; boshumin@cupes.edu.cn; 5 College of Medical Engineering, Xinxiang Medical University, Xinxiang 453000, China; 151036@xxmu.edu.cn

**Keywords:** metabolomics, exercise, muscle damage, blood biochemical analysis, salivary biomarker

## Abstract

Background: Exercise-induced muscle damage (EIMD) is the most common health risk in training. So far, EIMD diagnosis predominantly relies on blood biochemical analysis or medical imaging. EIMD prediction by using saliva shows great prospects in public fitness. Methods: A total of 18 participants performed high-intensity rowing training. Blood biochemical indicator and pain analyses indicated EIMD occurrence. Pseudo-targeted metabolomics techniques were utilized to analyze changes in salivary metabolites after exercise. Results: A total of 43 salivary metabolites significantly increased while 31 salivary metabolites significantly decreased after exercise. The upregulated metabolites were related to hormone secretion, antioxidation, and muscle repair. A partial least squares discriminant analysis model was established, and three potential salivary biomarkers for EIMD prediction were screened. The sensitivity and specificity of single biomarkers achieved more than 88.9% and 94.4% in classification of EIMD occurrence, respectively. The accuracy of classification increased to ~100% with multiple metabolites. Conclusion: Salivary metabolites significantly changed after high-intensity rowing training and EIMD occurrence. Some salivary metabolites exhibited similar trends with blood biochemical indicators. Salivary biomarkers have great prospects in EIMD prediction, and better performance was achieved with multiple salivary metabolites.

## 1. Introduction

Exercise plays a crucial role in maintaining physical health and preventing chronic diseases, effectively improving sleep quality and reducing the incidence of cardiovascular diseases and diabetes [1,2]. The World Health Organization recommends that adults engage in at least 150–300 min of moderate-intensity exercise or at least 75–150 min of high-intensity exercise per week [3]. Additionally, recent studies indicate that short-term high-intensity daily exercise, such as running, fast cycling, basketball, and soccer, is associated with a 22% reduction in mortality risk and a 20% reduction in cancer risk [4,5,6,7]. However, high-intensity exercise may also pose health risks [8], the most common of which is exercise-induced muscle damage (EIMD) [9]. High-intensity exercise, especially eccentric exercise, excessively stretches muscle cells, leading to the disruption of sarcomere ultrastructure [10]. Concurrently, increased metabolic stress in high-intensity exercise results in calcium overload and free radical accumulation, further damaging and lysing muscle cells [11]. The most typical symptoms of EIMD include delayed-onset muscle soreness (DOMS) and strength loss, which usually manifest 24 h post-exercise and persist for several days [12]. Additionally, EIMD is often accompanied by localized inflammation and prolonged training recovery [9]. Severe EIMD may induce rhabdomyolysis, resulting in myoglobinuria, hyperkalemia, hypocalcemia, and disseminated intravascular coagulation [13]. Therefore, an early diagnosis of EIMD is of great importance for minimizing exercise-related health risk.

So far, the most common method for EIMD diagnosis relies on blood biochemical analysis. Levels of creatine kinase (CK) and lactate dehydrogenase (LDH) in serum obviously increase with the occurrence of EIMD, and they have been used as serum indicators of EIMD [14,15]. The main reason is that when EIMD occurs, especially during high-intensity exercise such as eccentric exercise, muscle cells are excessively stretched, leading to the disruption of sarcomere ultrastructure. Furthermore, EIMD occurrence increases metabolic stress, resulting in calcium overload and free-radical accumulation, further damaging and lysing muscle cells. Therefore, myoglobin, C-reactive protein, cortisol, and testosterone levels in serum are related to EIMD [16]. Therefore, these serum indicators have been used for the auxiliary diagnosis of EIMD. Blood biochemical analysis has been employed in athlete training but is not suitable for public fitness. The main reason is that blood biochemical analysis requires professional technicians and biochemical analyzers [17]. Blood sampling also exerts physical and psychological stress, which may interfere with exercise performance [18].

Recently, some studies have reported non-invasive methods for EIMD diagnosis. Medical imaging methods, such as ultrasound imaging [19], magnetic resonance imaging [20], optical coherence tomography [21], and infrared observation [22], have been used to monitor muscle tissue changes. However, medical imaging equipment is expensive and cannot be easily operated. Compared to medical imaging, electromyographic signals are easier to measure and are associated with EIMD [23], but specific electromyographic indicators have not yet been reported. Self-diagnosis of EIMD is achieved using the combination of muscle strength assessment [24], but the significant decrease in muscle strength typically lags 24 h behind EIMD onset [25]. Therefore, further research is required to develop non-invasive methods for the early diagnosis of EIMD.

Saliva has been utilized for non-invasive disease diagnosis. Currently, saliva is mainly used in the diagnosis of oral diseases. Numerous micro RNAs, such as miR-200a and miR-31, have been employed in diagnosing oral squamous cell carcinoma (OSCC) [26]. Additionally, a recent study identified 25 salivary metabolites as potential biomarkers of OSCC [27]. Metabolites in saliva, such as butyrate, have been used to diagnose orthodontically induced external apical root resorption [28], while TLR4 mRNA has been applied for diagnosing periodontitis [29]. Furthermore, recent research has also reported the association of salivary metabolites with non-oral diseases. For instance, higher cortisol levels have been observed in the saliva of Parkinson’s disease patients [30]. Lower prolactin levels have been found in the saliva of lung and prostate cancer patients [31,32]. In diabetic patients, significantly elevated levels of salivary amylase have been observed [33]. Consequently, recent studies have focused on using salivary biomarkers for the diagnosis of non-oral diseases. Shu et al. reported that fucosylated N-/O-linked glycans serve as a biomarker for gastric cancer [34], and salivary pepsin has been utilized for diagnosing gastroesophageal reflux disease and laryngopharyngeal reflux [35,36,37,38]. Considering that various disease biomarkers are present in saliva, it is reasonable that biomarkers of EIMD might also be present in saliva.

A series of studies have demonstrated that exercise induces changes in salivary metabolites. Alzharani et al. reported that two days of soccer training resulted in significant changes in 27 salivary metabolites, with 22 being upregulated and 5 being downregulated [39]. Ra et al. further elucidated the physiological functions of metabolite changes [40]. In this study, three days of soccer training led to the significant upregulation of 10 salivary metabolites. These metabolites were mainly involved in glucose synthesis and the tricarboxylic acid cycle, supporting higher energy metabolism. In fact, short-term high-intensity exercise also obviously changed salivary metabolites. For instance, Pitti et al. found significant changes in 17 salivary metabolites after a soccer match (~90 min), with 14 being upregulated and 3 downregulated. Moreover, the trend of salivary metabolite changes varied with training methods. Compared to outfield players, goalkeepers exhibited unique changes in salivary metabolites post-match, e.g., an increase in formate and a decrease in isocaproate [41]. Notably, different from outfield player training, goalkeeper training involves rapid-response activities such as jumping, diving, and falling, which entails higher risks of muscle damage [42,43]. Therefore, unique metabolite changes in goalkeeper training might be related to EIMD. However, EIMD onset was not analyzed in the above studies, and salivary biomarker screening of EIMD has not been reported.

Therefore, this study aimed to clarify salivary metabolite changes after high-intensity exercise and screen potential salivary biomarkers for EIMD prediction. First, 18 untrained young male participants were recruited and subjected to high-intensity rowing exercise. Then, EIMD occurrence was determined by blood biochemical analysis. Following that, quasi-targeted metabolomics was employed to analyze the changes in and characteristics of salivary metabolites immediately after exercise, elucidating differential metabolites and metabolic pathways. The metabolites strongly correlated with blood biochemical indicators were also identified. Finally, potential salivary biomarkers for EIMD prediction were screened, and the performances of multiple salivary metabolites in EIMD prediction were evaluated.

This study first reveals salivary metabolite variation after high-intensity rowing training and proposes potential biomarkers for EIDM prediction. The results not only contribute to the interpretation of metabolite variation after high-intensity exercise but also provide a basis for the development of point-of-care device for EIMD prediction.

## 2. Material and Methods

### 2.1. Subject

A total of 18 healthy young male participants were recruited for this study. All participants were university students with a mean age of 23 years and an average BMI of 22.9 ± 1.8 kg·m^−2^ (Table 1). A questionnaire confirmed that none of the participants had regular exercise habits or previous experience with rowing training. To minimize exercise-related risks, all participants were free from cardiovascular and musculoskeletal diseases. To prevent the influence of oral diseases on salivary metabolites, none of the participants had periodontal diseases or other oral conditions, as determined by self-report. Additionally, none of the participants experienced bacterial or viral infections during the experiment because infection may induce pathological interference with CK levels. At rest, all participants exhibited normal levels of blood indicators of EIMD (CK: 55–170 U/L, LDH: 125–220 U/L). All the participants provided written informed consent before exercise, and approval was obtained from the ethics committee of the Beijing Institute of Technology [BIT-EC-H-2022143, approved on 11 August 2022].

### 2.2. Exercise Protocol

All participants were required to engage in rowing exercise using a magnetic resistance rowing machine (MRH3208A, Mobifitness Co., Shanghai, China). The maximum resistance was 32, and a high resistance of 30 was used in this study. The training goal was a total rowing distance of 3.0 km, requiring approximately 600 cycles of pulling and releasing. After every 200 m of rowing, the resistance was adjusted to level 1 for 30 s to provide a quick rest. During the rowing exercise, all the participants were asked to perform rapid pulling and slow releasing because releasing involves more eccentric exercise. Continuous encouragement was provided, and all the participants completed the set goal. Upon achieving the set goal, participants were asked to perform a vertical-jump, and the height of the jump was recorded to evaluate strength loss. DOMS was measured using the visual analog scale (VAS) at 24 h after exercise (Short for 24 h Post-Ex). Subjects were asked to mark a point on a VAS of 100 mm in length, where 0 mm signified “no pain” and 100 mm signified “extremely painful”.

### 2.3. Sample Collection and Preservation

Blood and saliva samples were collected from participants at two time points, including at rest (Pre-Ex) and immediately after exercise (Post-Ex). To avoid interference from blood sampling with exercise, the two sampling steps were not performed on the same day, but on two consecutive days. On the first day, participants were asked to have a standard breakfast at 8:00 am. Then, the participants rested for 1 h, and blood and saliva were sampled at around 9:00 am. On the second day, the participants had the same breakfast at 8:00 am. Then, the participants were asked to rest for 30 min and to perform rowing exercise. Blood and saliva samples were collected immediately after the exercise session at approximately 9:00 am. Blood sampling was conducted at the Beijing Institute of Technology Hospital, and the blood samples were preserved in serum separation tubes and analyzed on the same day. Saliva samples were self-collected by participants using saliva collection devices, and the pretreated saliva samples were stored at −80 °C for metabolite analysis.

### 2.4. Analysis of Blood Biochemical Markers and Salivary Metabolites

Two biochemical indicators of EIMD, including CK and LDH, were analyzed at Beijing Dian Diagnostics Laboratory. CK catalyzed the conversion of creatine and ATP to phosphocreatine and ADP, and the activity was quantitatively determined by measuring the rate of ADP production. LDH catalyzed the conversion of lactate and oxidized nicotinamide adenine dinucleotide (NAD^+^) to pyruvate and reduced nicotinamide adenine dinucleotide (NADH), and the activity was quantitatively determined by measuring the rate of NADH production.

Salivary metabolites were analyzed using quasi-targeted metabolomics with broad detection range and high accuracy. First, the saliva samples were centrifuged to remove cellular debris and impurities. Then, methanol was used to remove proteins in the saliva samples, and the metabolites were extracted and concentrated by lyophilization. Finally, liquid chromatography–mass spectrometry was utilized to measure metabolites. Metabolite detection was performed using the multiple reaction monitoring mode with a mass spectrometer (QTRAP 6500+, AB SCIEX, Marlborough, MA, USA) based on the Novogene database. Metabolite qualification was achieved by assessing Q3, while qualitative analysis was based on the retention time, Q1/Q3 ion pair information, and secondary spectral data.

### 2.5. Bioinformatics Analysis

The significance of blood indicator changes after exercise was analyzed by using the paired t-test function in Prism (version 10.2, GraphPad Software, USA). Salivary metabolite concentration was standardized before bioinformatics analysis. The standardization was performed by the ratio of metabolite concentration to creatinine concentration, which helped to mitigate the influence of salivary hydration status on metabolite concentrations [41].

Bioinformatics analysis was performed using the MetaboAnalyst 6.0 platform (www.metaboanalyst.ca (accessed on 15 July 2024)). The detailed analysis process is shown as follows:

(i) The overall changes in salivary metabolites after exercise were examined by using principal component analysis (PCA) and hierarchical clustering heatmaps. PCA was used to identify patterns of variation among samples. Hierarchical clustering showed the relationships between different samples and metabolites based on the similarity in metabolite profiles.
(ii) The salivary metabolites strongly correlated with CK and LDH were identified by using the pattern search module, and they are two important blood biochemical indicators of EIMD. The high correlation between salivary metabolites and CK/LDH was significant for understanding the physiological response to exercise, developing non-invasive diagnostic methods for EIMD, and exploring the body’s metabolic adaptation mechanisms.
(iii) Subsequently, differential metabolites after exercise were analyzed by using a paired *t*-test. The statistical test helped to determine which metabolites had significant changes between the Pre-Ex and Post-Ex groups. The enriched metabolic pathways were revealed by using the Kyoto Encyclopedia of Genes and Genomes (KEGG) module. KEGG is a comprehensive database that integrates genomic, chemical, and systemic functional information, which helped us to understand the biological functions and pathways associated with the differential metabolites.
(iv) A discriminant model for classification was established using partial least squares discriminant analysis (PLS-DA). The potential salivary biomarkers for EIMD prediction were screened by the operating characteristic curve (ROC). Based on the area under the curve (AUC) ranking, potential salivary biomarkers for EIMD prediction were screened. The prediction performances of multiple salivary metabolites were evaluated by using random forest, which contributed to determining the optimal number of metabolites for more accurate EIMD prediction.

## 3. Results

### 3.1. Blood Biochemical Indicators Analyses

**Figure 1:**
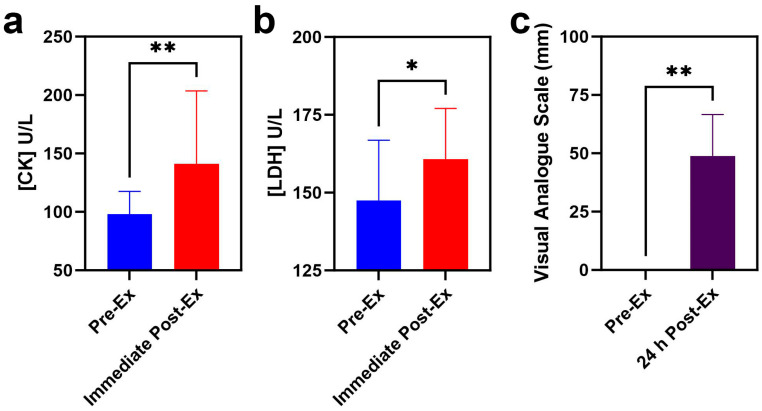
Serum indicators of EIMD and DOMS before and after rowing exercise ((**a**) CK; (**b**) LDH; (**c**) VAS score). (* indicates *p* < 0.05, while ** indicates *p* < 0.01).

In this study, 18 young male participants without regular exercise experience were asked to perform rowing exercise with high resistance, and all the participants successfully completed the set goal of exercise. CK levels increased from 98.1 ± 19.4 U/L to 141.2 ± 62.4 U/L with a *p* value of 0.004. Correspondingly, LDH levels rose from 149.8 ± 17.3 U/L to 162.8 ± 17.4 U/L with a *p* value of 0.0108 (Figure 1a,b). All the participants reported DOMS one day after training with a VAS value of ~50 mm, with a *p* value of 0.0003 (Figure 1c).

### 3.2. Changes in Salivary Metabolites After High-Intensity Rowing Training

**Figure 2:**
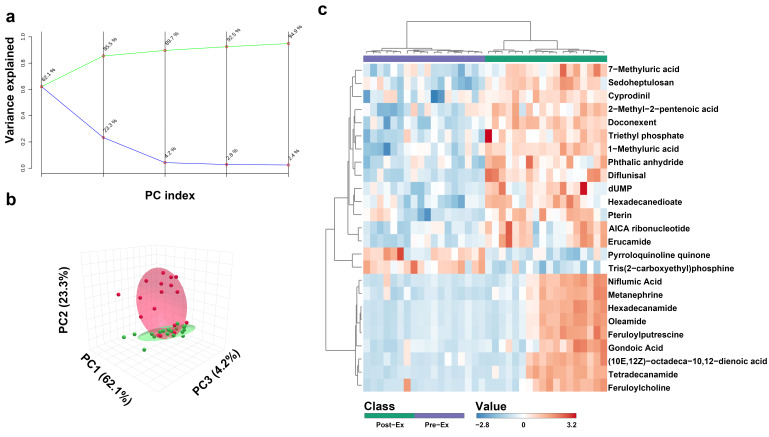
Overall changes in salivary metabolites after exercise ((**a**) PCA analysis with different principal components; (**b**) PCA analysis with three principal components (**c**) heatmap clustering analysis). (a Green line indicates cumulative variance explained while blue line indicates individual variance explained).

**Figure 3:**
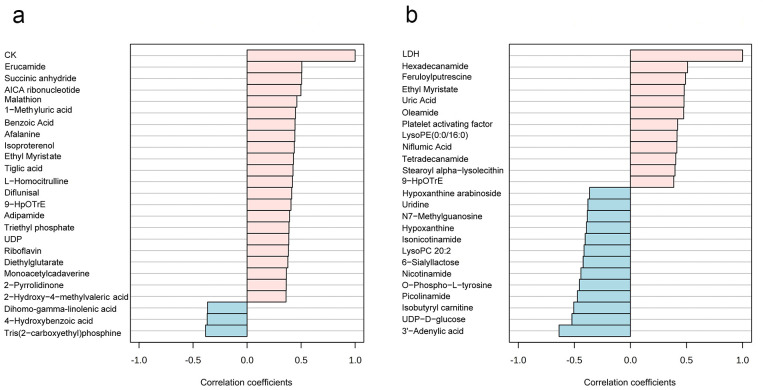
Relationships between salivary metabolites and blood indicators of EIMD ((**a**) salivary metabolites correlation with CK; (**b**) salivary metabolites correlation with LDH). (The red column represents positive correlation, while the blue column represents negative correlation).

A total of 594 metabolites were revealed in 36 saliva samples, indicating that more metabolites were observed in this study [39]. All the 36 saliva samples were visualized by PCA analysis, and three principal components collectively accounted for 89.7% of metabolite variation (Figure 2a,b). In the PCA score plot (Figure 2b), the green ellipse represents the samples of Pre-Ex, while the red ellipse delineates Immediate Post-Ex, demonstrating clear separation between groups. Notably, 18 samples in the Pre-Ex group are clustered in one region, while 18 samples in the Post-Ex group are clustered in another region (Figure 2b). Similar results were observed by cluster heatmap analysis (Figure 2c). All the 36 samples are clustered into two groups, which is completely consistent with the experimental grouping. Among these metabolites, the changes in some metabolites exhibit high correlation with CK and LDH (Figure 3). Erucamide is positively correlated with CK, while Tris(2-carboxyethyl)phosphine is negatively correlated with CK (Figure 3a). Hexadecanamide is positively correlated to LDH, while 3′-Adenylic acid is negatively correlated to LDH (Figure 3a).

**Figure 4:**
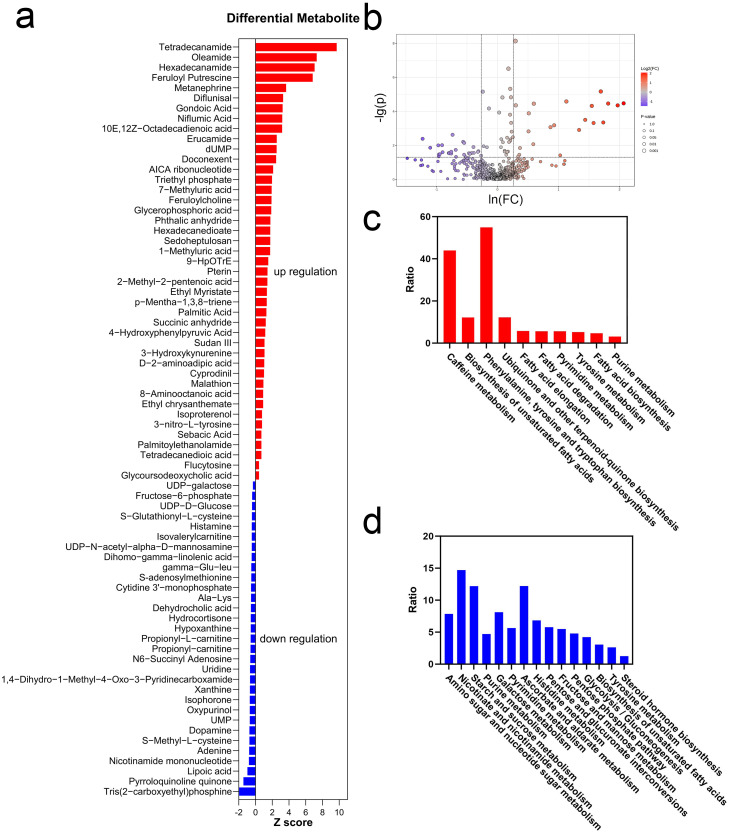
Differential metabolites and pathway analyses ((**a**) z-scores of differential metabolites; (**b**) volcano plot of differential metabolites; (**c**) enriched pathways of upregulated metabolites; (**d**) enriched pathways of downregulated metabolites).

A total of 74 metabolites exhibited significant changes immediately after exercise, with 43 metabolites being upregulated and 31 metabolites being downregulated (Figure 4a,b). According to z-scores, the top 10 upregulated metabolites were Tetradecanamide, Oleamide, Hexadecanamide, Feruloyl Putrescine, Metanephrine, Diflunisal, Gondoic Acid, Niflumic Acid, 10E,12Z-Octadecadienoic Acid, and Erucamide. According to z-scores, the top 10 downregulated metabolites were Tris(2-carboxyethyl)phosphine, Pyrroloquinoline Quinone, Lipoic Acid, Nicotinamide Mononucleotide, Adenine, S-Methyl-L-cysteine, Dopamine, UMP, Oxypurinol, and Isophorone.

Differential metabolites were due to the metabolic pathway changes after exercise, and they were illustrated by KEGG enrichment analysis (Figure 4c,d). The downregulated pathways were mainly related to carbohydrate metabolism. Similarly, pentose phosphate metabolism, starch and sucrose metabolism, and glycogen biosynthesis were significantly downregulated. In comparison, upregulated pathways were related to hormone secretion, antioxidation, and muscle repair. Two Methyluric acids in caffeine metabolism were upregulated. The biosynthesis of unsaturated fatty acids (UFAs) was upregulated. Additionally, the biosynthesis of terpene quinones was significantly increased. Phenylalanine, tyrosine, and tryptophan biosynthesis was markedly upregulated after exercise. Simultaneously, tyrosine metabolism was also upregulated. Both pyrimidine metabolism and purine metabolism upregulated.

### 3.3. Potential Saliva Biomarker Screening for EIMD Prediction

**Figure 5:**
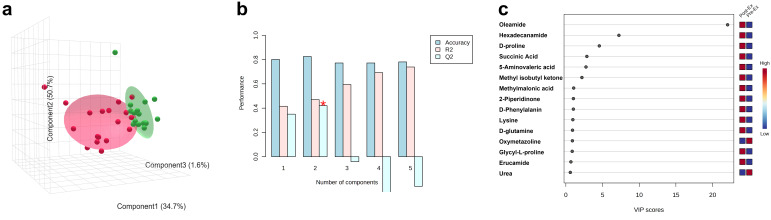
Discriminant analysis performed by using PLS-DA (**a**), prediction accuracy (**b**), and metabolites with high VIP values (**c**). (* indicates most highest Q2).

The discriminant analysis was performed by using PLS-DA, which is one of the dimensionality reduction methods used under labeled conditions. Two groups, i.e., the Pre-Ex (green ellipse) and Post-Ex groups (red ellipse), were clearly separated in three-dimensional space (Figure 5a). A discriminative model was generated to distinguish the two groups, and a mode accuracy of 83% was achieved by using two components (Figure 5b). The variable importance in projection (VIP) value was used to determine the importance of saliva metabolites in discriminant analysis. As shown in Figure 5c, the top 10 metabolites ranked by VIP scores included Oleamide, Hexadecanamide, D-proline, Succinic Acid, 5-Aminovaleric Acid, Methyl Isobutyl Ketone, Methylmalonic Acid, 2-Piperidinone, D-Phenylalanin, and Lysine.

**Figure 6:**
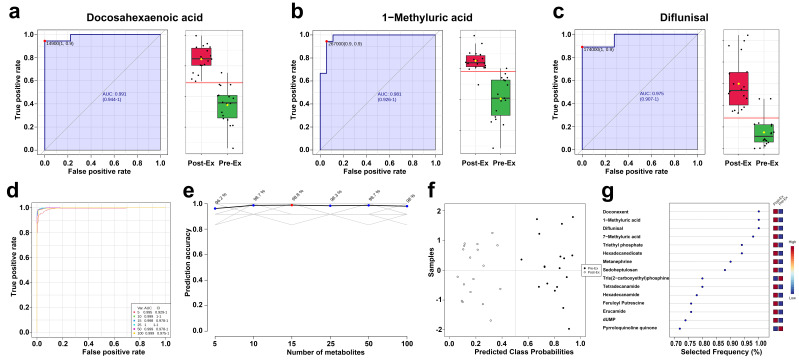
Prediction performance of salivary biomarkers ((**a**) AUC curve of Docosahexaenoic acid; (**b**) AUC curve of 1-Methyluric acid; (**c**) AUC curve of Diflunisal; (**d**) AUC curves of the combination of multiple metabolites; (**e**) relationship between prediction accuracy and the number of metabolites; (**f**) confusion matrix; (**g**) top ranking selected frequency of metabolites).

ROC was used to evaluate the prediction performance of salivary metabolites. Based on AUC, the top 3 salivary metabolites were Docosahexaenoic acid, 1-Methyluric acid, and Diflunisal (Figure 6a–c). Specifically, the specificity and sensitivity of the three salivary metabolites exceeded 88.9% and 94.4%, respectively. Additionally, the combination of multiple metabolites exhibited better performance in classification. As shown in Figure 6d, the AUC area increased with the number of saliva metabolites (Figure 6d), and the prediction accuracy for classification increased to 96.2% when using random forest with five metabolites (Figure 6e). Best performance was obtained when using 25 metabolites, and the confusion matrix indicated that classification accuracy reached ~100% (Figure 6f). For EIMD prediction using multiple metabolites, the metabolites with the highest frequency were Doconexent, 1-Methyluric acid, and Diflunisal.

## 4. Discussion

Serum CK and LDH levels significantly increased immediately after exercise. The elevations in CK and LDH were derived from muscle cell rupture and intracellular content release, which may be due to the release movement of rowing, which is concentric exercise. Previous studies have demonstrated that EIMD occurs easily in concentric exercise, followed by an immediately significant increase in CK and LDH. For example, Pokora et al. reported that the serum CK level significantly increased immediately following eccentric exercise, while it did not significantly change after concentric exercise [44]. Similarly, serum LDH levels significantly increased immediately after eccentric contraction [45]. Besides the increase in CK and LDH, the most common symptom of EIMD is DOMS. Therefore, both the significant elevations in serum indicators and obvious DOMS indicate EIMD occurrence after exercise in this study.

Previous studies mainly utilized resistance training to induce EIMD in specific muscle groups [46,47,48], whereas our research employed high-intensity rowing exercises. The choice of rowing exercise was due to four reasons. First, none of the participants had prior experience with rowing exercise, i.e., rowing exercise was an unfamiliar form of exercise. Additionally, the maximum resistance of the rowing machine was 32, while the resistance for rowing exercise was set at 30 in this study. Unfamiliar and challenging exercises are considered to increase the risk of EIMD occurrence. Secondly, rowing is a form of full-body exercise, with approximately 50% of power generated from legs, 30% from the torso, and 20% from arms [49]. All these muscles perform eccentric contractions during release movement, which are more likely to induce EIMD in several muscle groups, not limited to specific muscles. Thirdly, rowing exercise involves repetitive movements, requiring approximately 600 cycles of pulling and releasing. Repeated high-intensity movements may induce muscle fatigue and increase the risk of EIMD [11]. Finally, rowing exercise involves aerobic and resistance training, during which high oxygen consumption is required. A high oxygen consumption rate accelerates mitochondrial electron leakage, increasing the generation of free radicals, which increases the risk of EIMD [9].

Salivary metabolites were analyzed using quasi-targeted metabolomics. PCA analysis and cluster heatmap analysis demonstrated that rowing exercise induced obvious changes in salivary metabolites, and the changes appeared to have a similar trend [40]. Additionally, some metabolites exhibited similar variation trends to CK and LDH. So far, the correlation between salivary metabolites and serum biochemical indicators has not been reported. A high correlation may derive from the same mechanical and metabolic stress during exercise or insight into metabolic adaptation. The detailed mechanism remains in need of further research.

So far, only limited differential metabolites have been reported in previous studies. Hypoxanthine and Histamine were significantly downregulated, while 3-nitro-L-tyrosine was upregulated after rowing exercise, and a similar phenomenon was observed after soccer training in soccer players [41]. A decrease in S-Glutathionyl-L-cysteine and an increase in 4-Hydroxyphenylpyruvate after exercise was also observed in two previous studies [50,51]. In contrast, some metabolite changes were different from previous results. Xanthine was significantly downregulated after exercise in this study, but it exhibited an obvious increase after acute endurance exercise and resistance exercise [52,53]. The inconsistency in purine metabolite changes may be derived from variations in training duration [39]. Except for these metabolites, most differential metabolites have not previously been reported. So far, most research related to metabolomics in exercise and sports has only focused on the changes in serum and urinary metabolites [54]. This study contributes to deeply interpreting the effects of common exercise and sports on salivary metabolites.

The changes in salivary metabolites were derived from variations in metabolic pathways. Amino sugar and nucleotide sugar metabolism involves glucose consumption, and the downregulation may be due to the priority of glucose utilization for ATP production [55]. Caffeine metabolism was upregulated, which has been demonstrated to participate in free radical scavenging [56]. UFAs, which have been used as ergogenic aids for athletes [57], were also upregulated. The biosynthesis of terpene quinones was upregulated, and the most common terpene quinone was coenzyme Q, which is an antioxidant [50]. Furthermore, the upregulated amino acid metabolism pathways contribute to hormone and neurotransmitter secretion, e.g., Metanephrine [58]. Upregulated purine metabolism may be due to cell repair requiring more DNA synthesis [59].

In the PLS-DA model, more or less components decreased the accuracy, indicating that two components are optimal for group prediction. Referring to blood biochemical analysis, EIMD occurred in the Post-Ex group. Therefore, saliva metabolites are capable of indicating exercise, and they can be used to predict EIMD occurrence. According to AUC, the three metabolites, including Docosahexaenoic acid, 1-Methyluric acid, and Diflunisal, exhibited good performance in classification. Considering that EIMD only occurred in the Post-Ex group, these findings indicated that saliva metabolites hold great promise in EIMD prediction, and the three metabolites were considered potential biomarkers. Compared with single metabolites, the combination of multiple metabolites exhibited better performance in classification. The simultaneous detection of multiple saliva biomarkers is expected to more accurately predict EIMD.

So far, EIMD diagnosis mainly relies on blood biochemical analysis in athletes. Blood biochemical analysis requires invasive sampling, professional technicians, and expensive equipment, which are not suitable in public fitness. In fact, alternative EIMD diagnosis suitable for public fitness has not been reported. In this study, three potential salivary biomarkers for EIMD were primarily screened. Compared to blood sampling, saliva collection was non-invasive and characterized by easy operation, providing a foundation for the development of simple methods for EIMD prediction. Furthermore, these salivary biomarkers were small-molecule metabolites, and the rapid quantification could be realized by molecularly imprinted electrochemical sensors.

This study proposes potential biomarkers for EIMD prediction. Further validation should be performed using different exercise receipts. Despite the high correlation between metabolite changes and EIMD occurrence, a detailed mechanism for the correlation should be revealed. Furthermore, methods for the rapid detection of these metabolites should be developed, e.g., molecular imprinting, bioelectrochemistry or miniature mass spectrometry. More efforts are needed to develop point-of-care testing equipment for EIMD prediction.

## 5. Conclusions

High-intensity rowing exercise induced EIMD and resulted in significant changes in salivary metabolites. The upregulated metabolites were related to hormone synthesis, antioxidation, and muscle repair. Three potential salivary biomarkers were screened for EIMD prediction, and better prediction performance was obtained by using multiple salivary metabolites.

## Figures and Tables

**Table 1:** Physical indicators of participants.

Physical Indicators	Range	Average Value ± Standard Error
Height/cm	168–186	177.6 ± 4.6
Weight/kg	58–86	72.3 ± 7.0
BMI/kg·m^−2^	20.3–27.1	22.9 ± 1.8

## Data Availability

The metabolome data reported in this paper have been deposited in the OMIX, China National Center for Bioinformation/Beijing Institute of Genomics, Chinese Academy of Sciences (https://ngdc.cncb.ac.cn/omix/release/OMIX008903 (accessed on 5 February 2025)).
